# Resolution of dysglycaemia after treatment of monoclonal gammopathy of endocrine significance

**DOI:** 10.1093/ejendo/lvad138

**Published:** 2023-10-11

**Authors:** Bonnie Grant, Gowri Ratnayake, Claire L Williams, Anna Long, David J Halsall, Robert K Semple, James D Cavenagh, William M Drake, David S Church

**Affiliations:** Department of Endocrinology, St Bartholomew’s Hospital, Barts Health NHS Trust, London EC1A 7BE, United Kingdom; Department of Endocrinology, St Bartholomew’s Hospital, Barts Health NHS Trust, London EC1A 7BE, United Kingdom; Translational Health Sciences, Bristol Medical School, University of Bristol, Learning and Research, Southmead Hospital, Bristol BS10 5NB, United Kingdom; Translational Health Sciences, Bristol Medical School, University of Bristol, Learning and Research, Southmead Hospital, Bristol BS10 5NB, United Kingdom; Department of Clinical Biochemistry and Immunology, Cambridge University Hospitals NHS Foundation Trust, Cambridge CB2 0QQ, United Kingdom; Centre for Cardiovascular Science, Queen’s Medical Research Institute, University of Edinburgh, Edinburgh EH16 4TJ, United Kingdom; MRC Human Genetics Unit, Institute of Genetics and Cancer, The University of Edinburgh, Edinburgh EH4 2XU, United Kingdom; Department of Haematolo-Oncology, St Bartholomew’s Hospital, Barts Health NHS Trust, London EC1A 7BE, United Kingdom; Department of Endocrinology, St Bartholomew’s Hospital, Barts Health NHS Trust, London EC1A 7BE, United Kingdom; Department of Clinical Biochemistry and Immunology, Cambridge University Hospitals NHS Foundation Trust, Cambridge CB2 0QQ, United Kingdom

**Keywords:** insulin autoantibodies, hypoglycaemia, hyperinsulinaemia, plasma exchange, lenalidomide, myeloma, MGUS, smouldering myeloma

## Abstract

In very rare cases of monoclonal gammopathy, insulin-binding paraprotein can cause disabling hypoglycaemia. We report a 67-year-old man re-evaluated for hyperinsulinaemic hypoglycaemia that persisted despite distal pancreatectomy. He had no medical history of diabetes mellitus or autoimmune disease but was being monitored for an IgG kappa monoclonal gammopathy of undetermined significance. On glucose tolerance testing, hyperglycaemia occurred at 60 min (glucose 216 mg/dL) and hypoglycaemia at 300 min (52 mg/dL) concurrent with an apparent plasma insulin concentration of 52 850 pmol/L on immunoassay. Laboratory investigation revealed an IgG2 kappa with very high binding capacity but low affinity (Kd 1.43 × 10^−6^ mol/L) for insulin. The monoclonal gammopathy was restaged as smouldering myeloma not warranting plasma cell–directed therapy from a haematological standpoint. Plasma exchange reduced paraprotein levels and improved fasting capillary glucose concentrations. Lenalidomide was used to treat disabling hypoglycaemia, successfully depleting paraprotein and leading to resolution of symptoms.

**Significance**

In the last 5 decades, only rare, scattered patients with insulin-binding paraprotein in plasma cell dyscrasia have been reported. We present the clinical and biochemical characteristics of a man with disabling hypoglycaemia in whom a high-capacity insulin-binding paraprotein was identified. Using a suite of biochemical approaches, the insulin-binding antibody was confirmed to have the same characteristics as the pre-existing monoclonal gammopathy. We believe this is the first reported successful use of lenalidomide in smouldering myeloma to treat life-threatening dysglycaemia due to insulin-binding paraprotein.

## Introduction

Insulin autoimmune syndrome (IAS, Hirata disease) is a rare cause of hyperinsulinaemic hypoglycaemia characterised by insulin-binding autoantibodies in exogenous insulin-naïve individuals.^1,2^ In affected patients, antibody binding reduces the action of acutely secreted insulin and delays clearance of plasma insulin resulting in increased insulin concentration with raised insulin/C-peptide molar ratio.^1,3,4^ Insulin immunocomplexes act as reservoirs, with insulin–glucose mismatch leading to post-prandial and/or fasting hypoglycaemia. Even more rarely, plasma cell dyscrasia (PCD)-associated production of monoclonal antibodies/paraprotein with sufficient affinity and concentration to bind insulin and alter its kinetics has been reported.^5-17^ Both entities are characterised by very high plasma insulin concentrations but can vary in severity of hypoglycaemia at presentation. Laboratory investigation is not standardised, yet obtaining evidence that insulin-binding globulin is due to PCD rather than co-existence of IAS with coincidental asymptomatic PCD can have clinical utility. While IAS may be mild, and spontaneously resolve, disabling hypoglycaemia may persist in some patients and immunosuppressive regimens employed.^1^ In contrast, cytotoxic chemotherapy has been used in monoclonal insulin-binding antibody-associated multiple myeloma (MM).^8,12,15,16^ In 1 report, it was used largely unsuccessfully to treat hypoglycaemia in the context of monoclonal gammopathy of undetermined significance (MGUS).^10^ We now describe the clinical and laboratory investigation of a man with severe recurrent hypoglycaemia after pancreatectomy, who was ultimately confirmed as having an insulin-binding paraprotein. He experienced symptom resolution following treatment with lenalidomide and dexamethasone.

## Methods

### Insulin and C-peptide immunoassay

Plasma insulin and C-peptide were measured using the DiaSorin LIAISON XL chemiluminescence analyser. Plasma dilution for insulin analysis was undertaken using assay diluent.

### Insulin immunocomplex detection

Polyethylene glycol (PEG) precipitation studies of plasma, and gel filtration chromatography (GFC) of plasma post addition of recombinant human insulin, were undertaken as previously published.^18^

### Radioligand binding assays

Insulin-binding affinity by antibody was assessed in a 10-fold dilution with anti-insulin antibody negative serum, with immune complexes precipitated using a 50:50 mixture of protein A Sepharose and protein G Sepharose as previously outlined.^1,19,20^ Kd (mol/L) was calculated by non-linear regression analysis using a 1-site model (*R*^2^ value .98), assuming equal antibody binding by labelled ([125-I]-A14) and unlabelled insulin.

Insulin-binding antibody class was determined using competitive radioligand binding assay described previously.^1,21^ Complexes of 125I-insulin antibody were precipitated using either glycine-blocked protein A Sepharose (GE Healthcare), ethanolamine-blocked protein G Sepharose (GE Healthcare), IgA agarose (Sigma), or IgM agarose (Sigma).^22^

Insulin-binding IgG subclass was determined by radioligand binding assay using [125-I]-A14 insulin and biotinylated IgG subclass-specific mouse anti-human monoclonal antibodies (*BD Biosciences, San Diego, United States; **Invitrogen, Thermo Fisher, CA, United States) bound by Streptavidin-Sepharose beads (Sigma-Aldrich, Dorset, United Kingdom)^23,24^ described previously. Mouse anti-human IgG subclass antibodies used were IgG1 (clone G17-1*), IgG2 (clone G18-21*), IgG3 (clone HP6047**), and IgG4 (clone JDC-14*). Non-specific binding was determined by subtraction of mean counts per minute (CPM) of a mouse anti-rat IgM monoclonal antibody (clone G53-238*).

### Electrophoresis and light chain capture

Capillary zone electrophoresis was undertaken using the Helena Biosciences V8 system. Insulin-binding light chain was determined using the PureProteome™ Kappa Ig Binder and Lambda Ig Binder Magnetic Beads as directed by manufacturer's instructions. Patient triplicate mean results were compared with results from an IAS sample used as positive control and an anti-insulin antibody-negative control sample.

### Case presentation

A 67-year-old man was referred with a 10-year history of episodic loss of consciousness preceded by sweating, palpitation, fatigue, and intense hunger, resolving with refined carbohydrates. Previously, evaluation at another centre confirmed fasting hyperinsulinaemic hypoglycaemia and a possible Ga-68 DOTATATE-avid pancreatic lesion. He underwent distal pancreatectomy for suspected insulinoma; histology showed an 8 mm nodule without evidence of an endocrine tumour. Four years later, he was diagnosed with MGUS and monitored expectantly.^25^ Hypoglycaemia symptoms worsened during fasting and postprandially with hypoglycaemic unawareness ultimately developing. He had never been prescribed insulin or oral hypoglycaemic agents and had no family history of diabetes mellitus or autoimmune conditions. His body mass index was 32.2 kg/m^2^; examination was otherwise unremarkable.

**Figure 1.**
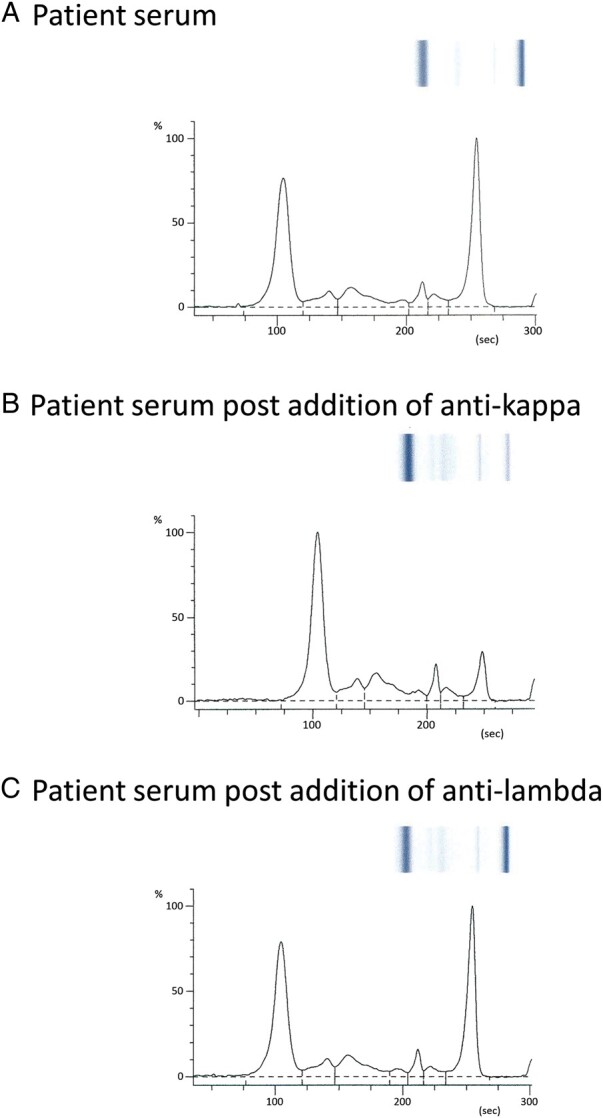
Capillary zone electrophoresis pre/post light chain capture. Results of capillary zone electrophoresis of serum (A), following addition of anti-kappa (B), and following addition of anti-lambda (C). Results were consistent with a monoclonal band, which was reduced with anti-kappa but not anti-lambda.

Investigations revealed IgG of 32.07 (reference interval 6.00-16.00) g/L with kappa light chain of 41.0 (3.3-19.4) mg/L. Lambda light chain, IgA, IgM, and total protein concentrations were within respective reference intervals. Serum quantification of IgG subclasses revealed IgG1 3.97 (3.20-10.20) g/L, IgG2 15.10 (1.20-6.60) g/L, IgG3 .07 (.20-1.90) g/L, and IgG4 .37 (.00-1.30) g/L. Serum protein electrophoresis confirmed a monoclonal band (Figure 1A) of 21 g/L, which was typed as IgG kappa. Low-dose computerised tomography skeletal survey did not reveal any lytic lesions, and bone marrow trephine showed 30% plasma cells, meeting criteria for smouldering myeloma (SM) not warranting treatment.^25^

**Figure 2.**
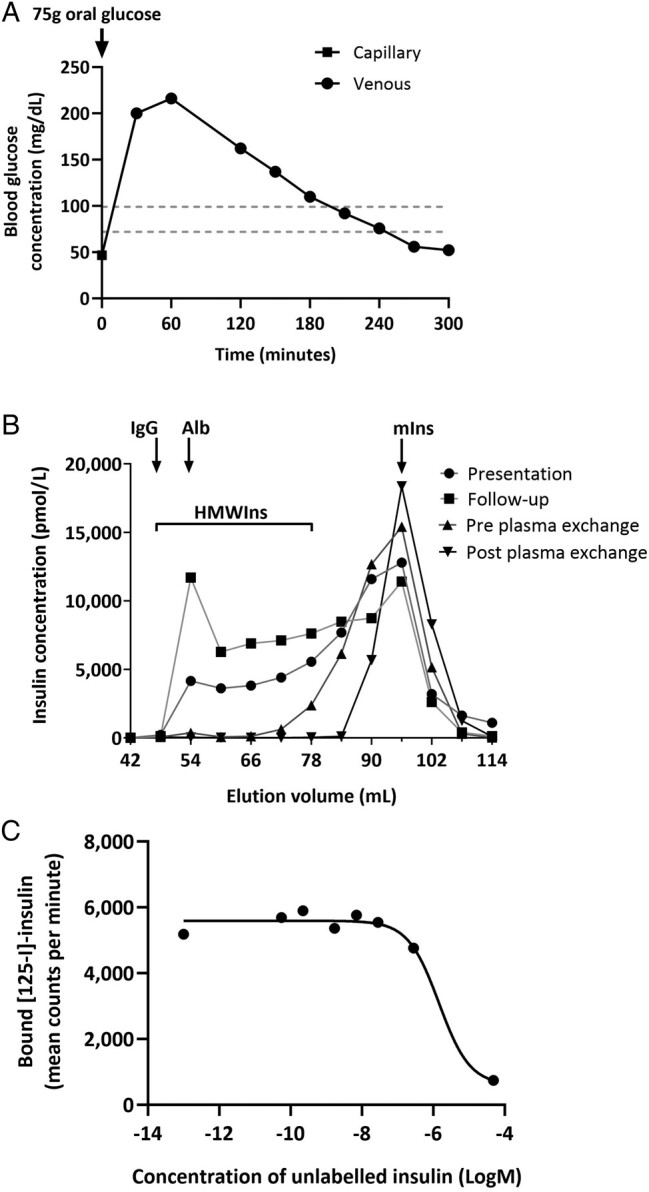
Prolonged oral glucose tolerance test (A). Fasting capillary blood glucose measured pre, and venous blood glucose measured post, 75 g oral glucose load. (Venous sample collected at time 0 was unsuitable for analysis due to haemolysis.) Gel filtration chromatography of plasma post addition of synthetic human insulin (B). Results from presentation, follow-up, pre plasma exchange, and post plasma exchange are shown. Elution volumes of immunoglobulin G (IgG), albumin (Alb), and monomeric insulin (mIns) are shown (molecular weights 150, 66.5, and 5.8 kDa, respectively). High-molecular-weight insulin (HMWIns) is antibody-bound insulin that elutes with immunoglobulin, and the slur between peaks is produced by dissociation of immunocomplexes during filtration. Radioligand binding assay (C). Data were consistent with an antibody of high capacity and low affinity (Kd 1.43 × 10^−6^ mol/L) to bind insulin.

He developed symptomatic hypoglycaemia after 10 h of a supervised fast with venous plasma glucose 36 mg/dL. A prolonged 75 g oral glucose tolerance test (OGTT) showed hyperglycaemia at 60 min (glucose 216 mg/dL) and hypoglycaemia at 300 min (52 mg/dL) (Figure 2A). Immunoassay revealed an insulin concentration of 52 850 pmol/L and C-peptide immunoreactivity of 5160 pmol/L. The degree of hyperinsulinaemia and high insulin/C-peptide molar ratio prompted studies that identified low recovery of insulin and C-peptide immunoreactivity following PEG precipitation (<.5%, reference limit >91%; 11%, reference limit >134%, respectively) consistent with high-molecular-weight immunoreactive components. Serum anti-insulin IgG was 181 (0-5) mg/L, and GFC confirmed antibodies with high insulin-binding capacity (Figure 2B) as the cause of hypoglycaemia. Radioligand binding studies (RBS) confirmed low affinity (Kd 1.43 × 10^−6^ mol/L) anti-insulin antibodies (Figure 2C).

**Figure 3.**
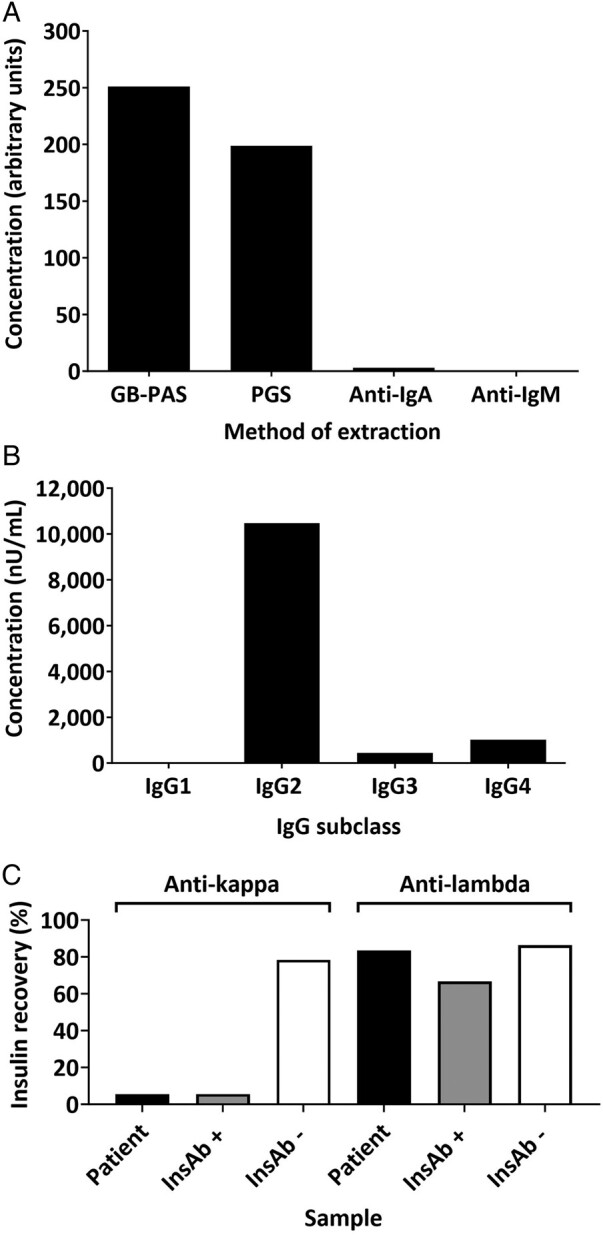
Insulin binding by antibody class (A). Results are expressed as arbitrary units (AU) derived from a logarithmic standard curve. Data demonstrated most insulin binding using protein A Sepharose (GB-PAS) and protein G Sepharose (PGS), consistent with anti-insulin IgG. Insulin binding by IgG subclass (B). Results are expressed as nano unit of insulin bound per millilitre of serum (nU/mL). Data are consistent with anti-insulin IgG2. Insulin recovery following light chain capture (C). Results expressed as percentage recovery of insulin immunoreactivity (%). Depletion of paraprotein with anti-kappa but not anti-lambda was confirmed with capillary zone electrophoresis (Figure 1B and C). InsAb +, positive control; InsAb -, negative control. Patient results are consistent with anti-insulin kappa.

Given the SM, determining whether the anti-insulin antibodies were due to IAS or PCD would direct management. Radioligand binding studies and light chain capture assays confirmed the principal insulin-binding globulin was IgG (Figure 3A) of IgG2 subclass (Figure 3B) and kappa light chain (Figure 3C) consistent with the M-protein.

Due to the COVID-19 pandemic, he was unable to attend follow-up appointments so was speculatively prescribed prednisolone (20-40 mg daily). A weight increase of 6.5 kg ensued, and recurrent hypoglycaemia with loss of consciousness persisted.

Smouldering myeloma reassessment 12 months later revealed no indication to treat from a haematological perspective. Paraprotein increased to 25 g/L and anti-insulin IgG to 242 mg/L with follow-up GFC results confirming a further rise in insulin-binding capacity (Figure 2B). Given ongoing life-threatening hypoglycaemia and inefficacy of prednisolone, the patient agreed to a trial of plasma cell–directed therapy (PCDT), namely lenalidomide 25 mg daily for 21/28-day cycle with dexamethasone 40 mg once weekly. Non-neutropenic sepsis developed within a week, and attempted dose reduction to lenalidomide 20 mg once daily resulted in a second similar hospital presentation.

To assess the potential benefit of insulin-binding antibody depletion, he underwent a 3-day course of plasma exchange.^1^ Prior to this, fasting capillary blood glucose (FCBG) ranged from 54 to 65 mg/dL, paraprotein level 16 g/L with anti-insulin IgG 159 mg/L, and some reduction in insulin-binding capacity (Figure 2B). Following plasma exchange, paraprotein fell to 2 g/L with equivocal residual insulin binding by day 2 (Figure 2B) and increased FCBG ranging from 92 to 153 mg/dL, providing proof of concept and support for ongoing PCDT.

Reduced dose lenalidomide 5 mg once daily for 21/28-day cycle was commenced, and improvement in glycaemic control followed within 3 months with FCBGs ranging from 67 to 110 mg/dL. This regimen has continued for over 18 months with no side effects and no hypoglycaemic symptoms.

## Discussion

Reports of hypoglycaemia caused by insulin-binding antibodies secreted in PCD are very rare. Hypoglycaemia may be postprandial, postabsorptive, or fasting, and, as in our patient, symptoms can be disabling. The very high plasma insulin concentration, increased insulin/C-peptide molar ratio, and positive anti-insulin IgG result were indistinguishable from that of IAS.^1^ Detectable C-peptide immunoreactivity concurrent in a hypoglycaemic sample was of high molecular weight and could represent antibody-bound proinsulin cross-reactivity in immunoassay.^1,26^

The investigative approach relied upon anti-insulin antibody assays not typically available outside a specialist laboratory, including GFC, RBS, and light chain capture assays. Results confirmed the presence of an anti-insulin antibody with a high capacity to bind insulin with low affinity, consistent with descriptions in other reports of monoclonal gammopathy with dysglycaemia.^10-16^ Binding affinity was lower than previously demonstrated in an IAS cohort using the same methodology.^1^ In IAS, autoantibodies are typically polyclonal but may be monoclonal.^27-31^ They are most commonly of the IgG class without predominance of a particular subclass or light chain.^32^ Further examination in this case confirmed the insulin-binding antibody to be IgG2 kappa, consistent with the paraprotein and increased serum concentration of IgG2. In the context of severe dysglycaemia requiring clinical intervention, discriminating the 2 entities had important management implications: although aberrant insulin kinetics are induced by a similar mechanism in both polyclonal IAS and monoclonal insulin binding, autoantibody production in IAS may be self-limiting, while in PCD, targeted PCDT is needed for antibody depletion.

Following initial side effects that followed initiation of PCDT, plasma exchange provided proof of principle that antibody depletion would improve glucose control and lead to symptomatic improvement. Lenalidomide was recommenced at a lower dose that resulted in resolution of symptoms and the absence of side effects. Although lenalidomide use with dexamethasone has been reported in MM and insulin-binding antibodies,^16^ we believe this is the first reported successful use of combination therapy in SM with insulin-binding paraprotein, whereby disabling hypoglycaemia directed therapy.
